# Recognition and Work in the Platform Economy: a Normative Reconstruction

**DOI:** 10.1007/s40926-021-00172-2

**Published:** 2021-03-20

**Authors:** Max Visser, Thomas C. Arnold

**Affiliations:** Institute for Management Research, Radboud University, PO Box 9108, 6500 HK Nijmegen, The Netherlands

**Keywords:** Recognition theory, Radical humanism, Normative reconstruction, Neoliberalism, Platform economy

## Abstract

The rise of the platform economy in the past two decades (and neoliberal capitalist expansion and crises more in general), have on the whole negatively affected working conditions, leading to growing concerns about the “human side” of organizations. To address these concerns, the purpose of this paper is to apply Axel Honneth’s recognition theory and method of normative reconstruction to working conditions in the platform economy. The paper concludes that the ways in which platform organizations function constitutes a normative paradox, promising flexibility and autonomy while at the same time creating working conditions that undercut these promises. The paper ends with a critical discussion of Honneth’s approach, possible supplementing ideas and further lines of future research.

## Introduction

The movie *Sorry We Missed You* depicts the lives of Ricky and Abby. Since the economic crisis, they struggle to make ends meet and provide for their two children. In the opening scene, Ricky has a job interview to become – as the recruiting manager describes it – a “driver-owner-franchisee,” which is essentially a self-employed parcel deliverer. The key terms of the working relationship appear rather promising. Rather than being hired, Ricky comes “on board.” He does not work for the company, but “with” the company. There is no employment contract nor wages, but “fees.” The manager describes the work as being “the master of your own destiny,” and promises him a working environment of freedom, choice and autonomy. In addition, the brochure promises astronomical turnovers of over a £100.000 in a matter of years. As the movie progresses, however, the true working conditions reveal themselves. Ricky and the other drivers are being bossed around like regular employees and bad behavior or absence – even under the worst of circumstances – results in significant penalty payments and potential termination of the working relationship. On top of that, the costs and risks of the job, such as renting the delivery van, damages or traffic fines, are borne by the drivers themselves.

While still a work of fiction, the movie seems to adequately capture the degradation and precarization of working conditions in the so-called platform economy of the past decades (and under neoliberal capitalist conditions more general, e.g., Davies 2016; Peck 2013). From a (radical) humanist perspective, these developments make a rethinking of notions of human dignity and respect in organizations both necessary and opportune. Historically, the “human side” of organizations first received attention in the Hawthorne studies before World War II. In the three decades after World War II, it became a central concern of the “human relations” school of Maslow, McGregor, Argyris and others, who sought ways to more fully and harmoniously integrate the needs of both employees and organizations than was possible under the then prevailing Scientific Management (Carson 2005; Lussier 2019). In the 1970s, concerns for the “human side” of organizations were taken up by radical humanists in the critical tradition of the Frankfurt School of Horkheimer, Adorno, Marcuse, Habermas and others, which radical humanism in its turn was subsumed under the emerging Critical Management Studies (CMS) of the 1990s and thereafter (Aktouf 1992; Alvesson and Willmott 1992; Scherer 2009).

Although thus evolving and changing over time, research on the “human side” of organizations in general has been concerned with respect for and recognition of employees as full human beings. Thus, McGregor’s (1960) Theory Y assumptions held that employees do not inherently dislike work and responsibilities and are capable of exercising self-direction and self-control towards attainment of organizational goals, provided that they are properly recognized in their ego and self-actualization needs. Maslow (1965: 17-33) proposed 36 assumptions about human nature that he believed were necessary for Theory Y to work, for example that “everyone prefers to feel important, needed, useful, successful, proud, respected, rather than unimportant, interchangeable anonymous, wasted, unused, expendable, disrespected…; preference for being a whole person and not a part, not a thing or an implement, or tool, or ‘hand’.”

Within radical humanism, respect and recognition are central elements in the work of Axel Honneth, a German social philosopher who is generally regarded as the most prominent representative of the current generation of Frankfurt School scholars (Anderson 2011; Keucheyan 2014; Outhwaite 2009; Zurn 2015). While still largely bypassed by CMS scholars (exceptions are Alvesson and Willmott 2012; Scherer 2009; Willmott 2020), in the past decades Honneth’s ideas on recognition have been brought to bear on a diversity of fields and subjects in management and organization studies (hereafter MOS), such as business ethics (Islam 2012; Visser 2019), human resource management (Islam 2013; Klikauer 2016; Tweedie et al. 2019; Varul 2005; Voswinkel 2011), management accounting (Tweedie 2018), organizational behavior (Connolly 2016; Holtgrewe 2001; Mokatef 2019; Petersen and Willig 2004; Sebrechts et al. 2019) and “meaningful” work (Bailey et al. 2019; Tweedie 2017; Tweedie and Holley 2016). In all these studies, Honneth’s recognition theory serves as a generally suitable vehicle for critiquing the instrumentalized and commodified nature of work under current economic conditions.

In this paper, our purpose is to use recognition theory in two ways that are largely novel to the field of MOS. First, we extend Honneth’s theory to an analysis and critique of working conditions in the platform economy. Second, in this analysis and critique we explicitly use Honneth’s method of normative reconstruction. Towards this purpose, in the second section of this paper, we discuss the basic tenets of Honneth’s recognition theory and his method of normative reconstruction. In the third section the platform economy and the place of work therein are described and normatively reconstructed, while in the final section of this paper we critically engage with Honneth’s theory itself and draw some conclusions.

## Honneth: Recognition Theory and Normative Reconstruction

Fundamental to Honneth’s social philosophy is a conception of freedom as intersubjectively constituted through mutual recognition. In order to achieve self-realization and develop a positive identity it is essential for persons to be recognized by other persons:

The point of departure for a social theory of this sort has to be the basic claim on which the pragmatist Mead and the early Hegel are agreed in principle: the reproduction of social life is governed by the imperative of mutual recognition, because one can develop a practical relation-to-self only when one has learned to view oneself, from the normative perspective of one's partners in interaction, as their social addressee (Honneth 1995: 92).

For Honneth, this intersubjective freedom becomes social freedom when extended to societal “institutions of recognition… bundles of behavioral norms that ‘objectively’ integrate individuals’ aims” (Honneth 2014: 45), like the family, the economy and the state.

Honneth (1995, 2007b) distinguishes three fundamental patterns in recognition, necessary for a person’s development of a positive relation-to-self. The first pattern, love, refers to the physical, emotional and affective presence of proximal significant others (family, close friends) in the course of one’s life, especially in childhood; as such, it goes beyond a contemporary, narrow definition of intimate love between two partners*.* In terms of consequences, this pattern contributes to basic self-confidence, which forms the basis for the development of other forms of self-respect and self-worth.

The second pattern, rights, refers to the acknowledgment of persons as autonomous and independent members of society, i.e. as bearers of equal legal, social and political rights. This pattern thus depends less on the physical presence and emotional commitment of others, but has a more rational character and depends on the institutional qualities of society. This pattern contributes to the development of self-respect.

The third pattern, solidarity, refers to the positive evaluation of personal traits and abilities by others. It presupposes what Honneth calls an “intersubjective value-horizon”, i.e. it requires that persons value these traits and abilities as something positive, in the absence of which one cannot truly recognize “the significance or contribution of [one’s] qualities for the life of the other” (Honneth 1995: 121). This pattern contributes to the development of self-esteem, the positive evaluation of one’s own capacities and achievements.

From these patterns of recognition, Honneth (1995, 2007b) deduces moral obligations that confer legitimate rights on persons, leading to normative expectations to be treated in accordance with these obligations. Recognition through love involves, for instance, the protection against physical and psychological abuse in primary relationships. Recognition through rights involves the protection against structural exclusion, the denial of legal, social and political rights to fully participate in society. Recognition through solidarity involves the protection against a denial or depreciation of persons’ contributions to society, based on traits, ways of life or other attributes of those persons. Violations of these normative expectations constitute instances of misrecognition and disrespect, which may inflict real social and psychological harm and pain on the persons subjected to it. The identification and alleviation of these instances constitute the normative core of Honneth’ critical approach (Anderson 2011; Outhwaite 2009).

Honneth (2007b, 2014) extends the patterns of recognition to the level of societal institutions by looking how moral obligations and normative expectations accrue from, for example, legal definitions, political representation, public policy and material redistribution. For acts of institutional recognition to be successful, it is required that they be positive (i.e. giving all subjects the ability to relate positively to themselves, thus excluding discriminatory classifications that would deny a certain value to a subject or group), credible (i.e., effectively leading to the strengthening of the self-image of its addressees), and contrastive (i.e., bestowing upon its addressee a sense of distinction) (Honneth 2007a).

Different from previous Frankfurt grand meta-narratives like the “eclipse of reason” or “colonization of the life-world,” Honneth directly turns to normative expectations that are available in society itself, and through his method of normative reconstruction he intends to uncover the existence of social, legal and economic practices that govern the differential realization of the norms underlying these expectations (Anderson 2011; Honneth and Sutterlüty 2011). More specifically, in two steps this method first entails the uncovering of general norms and values governing a certain institutional domain of society (e.g., family, economy), together with the more specific promises that accrue from them. Second, the method entails the identification of institutional practices that differentially affect the realization of these norms, values and promises inherent in that domain. When these norms are adequately realized, Honneth speaks of “moral progress,” but when these norms are not sufficiently realized, he speaks of “normative misdevelopment” (Curty 2020: 1341; Honneth 2014). When those institutional practices are seen as even preventing the realization of these norms, Honneth speaks of normative contradictions or paradoxes, whereby a “contradiction is paradoxical when, precisely through the attempt to realize such a [normative] intention, the probability of realizing it is decreased” (Hartmann and Honneth 2006: 47; Honneth 2004).

In a normative reconstruction along these lines of the socioeconomic developments in the Western capitalist countries since World War II, as a first step Honneth observes how in the decades after 1945 capitalism became firmly regulated and circumscribed by the national states, giving rise to a “normative surplus” feeding expectations of increasing individual autonomy, more extensive protection of legal and political rights and higher (material and immaterial) rewards for individual achievements; in all these areas Honneth discerns good moral progress, in particular in the era between the late 1960s to the early 1980s (Hartmann and Honneth 2006: 43; Zurn 2015). This changed, however, when in the course of the 1980s and thereafter this state-regulated capitalism gradually gave way to current neoliberal capitalism. At the state level, this form of capitalism is disorganized, reflecting trends of globalization, internationalization of finance flows and a decreasing role of state welfare rules and safeguards. At the firm level, this form of capitalism is predominantly shareholder oriented, valuing financial interests over those of other stakeholders (such as workers and consumers). At the individual level, this form of capitalism embraces flexibility and networking, requiring

no longer the ability to efficiently fulfill hierarchically determined parameters within a large enterprise… [but] the readiness to self-responsibly bring one’s own abilities and emotional resources to bear in the service of individualized projects. In this way, the worker becomes an “entreployee” or himself an entrepreneur; no longer induced to participate in capitalist practices by external compulsion or incentives, he is in a sense self-motivated (Hartmann and Honneth 2006: 45; Honneth 2014).

As a second step in his normative reconstruction, Honneth inquires how the institutional practices inherent in neoliberal capitalism affect the realization of these norms and promises of entrepreneurialism, flexibility and involvement and finds that they amount to “organized self-realization” (Honneth 2004) and “recognition as ideology” (Honneth 2007a). The ideals of entrepreneurialism and self-realization are not sustained by appropriate material conditions; organizations only allow for these ideals in so far as they conform to organizational goals and performance criteria, thus compelling workers “to feign initiative, flexibility, and talents in places where there are no roots for such values” (Honneth 2007a: 346; Islam 2012). Furthermore, features of personal identity development are turned into, even commodified as, organizational efficiency enhancing expectations, exhorting workers to continuously “sell themselves” and to be “authentic,” although always within the cultural parameters the organization proscribes (Honneth 2008: 83; Petersen and Willig 2004; Voswinkel 2011). This reversal of neoliberal norms and promises thus amounts to a normative paradox, which in the longer run is conducive to symptoms of “inner emptiness, of feeling oneself to be superfluous, and of absence of purpose” among the supposedly “entreployees” (Honneth 2004: 467; Honneth 2007a; Willmott 2020).

It is within this greater remit of the transition from state-regulated to neoliberal capitalism that we now turn to work in the platform economy and the normative reconstruction thereof.

## A Normative Reconstruction of Work in the Platform Economy

A normative reconstruction of platform work requires as a *first step* the uncovering of general norms and values governing the platform economy, together with the more specific promises that accrue from them. A starting point for uncovering these norms and values can be found in the key elements that characterize digital platforms as a novel category of enterprises, which utilize digital technologies to provide intermediation services and which create a distinctly new way in which individuals interact, consume and, most notably, work, with three characteristics: multi-sidedness, cross-side network effects, and the ability to drastically reduce transaction costs.

The first characteristic, multi-sidedness, is considered by many scholars as a defining feature of digital platforms (Evans 2008; Rochet and Tirole 2006; Wismer et al. 2017). Platforms perform intermediation services to various distinct “sides” of the market (e.g. consumers, producers), and this role as a mere “interaction facilitator” has two important consequences for the position of these market participants. First, platform work is executed on a solo self-employed basis. Platform workers do not fall under the general responsibility of the platform on which they operate, but merely perform tasks which are curated by the platform. This means that not the platform, but the workers themselves bear key health, commercial and occupational risks. Second, digital platforms are capable of exerting influence on each respective side of the market that they connect, which renders them potentially very powerful players, capable of influencing key market conditions. Whereas some digital platforms refrain from exercising such influence (“mere marketplaces”), others more expressly exercise control over the terms and conditions of the transactions on the platform. Uber is a good example, as it determines the main aspect of the transaction: the price of the ride (Chen and Sheldon 2015). However, as the influence of platforms on these conditions grows, the argument for qualifying them as “mere interaction facilitators” loses credibility. This is illustrated by the many court battles of Uber drivers and other platform workers seeking a legal qualification as employee rather than solo self-employed.

The second characteristic commonly associated with platforms is the existence of cross-side network effects (Evans 2008; Jullien 2005; Parker and Van Alstyne 2005; Rysman 2009). Since platforms, again, connect various distinct sides of the market, their value for platform users largely lies in the network of users to which they provides access (Zhu and Iansiti 2019). Direct network effects relate to same-side interactions, while indirect network effects reflect the value of cross-side interactions (Evans 2008; Jullien 2005). The latter are often deemed to produce so-called “feedback loops”, a feature to which many ascribe the ability of digital platforms to rapidly gain a large degree of market power and become “a winner who takes it all” (Zhu and Iansiti 2019), which function as follows. If the platform is able to increase demand for its intermediation on side A of the market, users on side B of the market will be drawn to the platform as well. After all, the value of the platform for side B-users lies in their ability to access a large user-base on side A of the market. Under the right circumstances, this process repeats itself, each increase in size of the user-base on one side resulting in an increase on the opposite side. At a certain tipping point, any alternative to that platform becomes irrelevant, since the majority of users is locked into the network of the incumbent platform (Prud’homme 2019).

The third characteristic pertains to the platforms’ ability to drastically reduce transaction costs. Starting as a mere “read-only” source of information, from the start of this century onwards the internet developed into a highly interactive medium that can be accurately molded to fit a great variety of purposes. It is this transformation into what has been called the “Web 2.0″ that seems to have unlocked the true potential of the platform model (Helmond 2015). Digital platforms have created multi-sided networks, which are made accessible via simple and intuitive mobile applications and thus drastically reduce search and information costs for consumers (Drahokoupil and Piasna 2017). Simultaneously, they increase heterogeneity of supply, since suppliers from across the globe can connect to the network and offer their tailor-made services. Consumers can easily determine quality and reliability of offerings through the detailed review and rating systems. Furthermore, digital platforms reduce efforts to conclude a transaction, by offering fluent payment solutions.

These characteristics of multi-sidedness, network effects and the ability to reduce transaction costs already contain a first clue for the normative promises enclosed in the platform model. Since workers essentially function as self-employed entrepreneurs that fulfil the function of independent service supplier to users on the other side of the platform, they are promised full control over the conditions of work, such as the number of working hours, working time schedules and the type of work they want to perform. Moreover, since a platform (in theory) merely facilitates a transaction between demand and supply, platform workers are free to choose the customers they serve. This also implies that platform workers are able to offer services across a variety of platforms, further increasing their freedom to shape material working conditions. Finally, these platforms offer vast networks of agents to interact with, at minimal cost. The digital platform model thus appears to be the ultimate device to realize the ideal of the entrepreneurial self, as it is built on the promise of practically borderless flexibility, self-management and autonomy.

The normative promises of a flexible workspace with high levels of worker autonomy also surface in practice. A fitting example is the “Owner Driver Franchise” (ODF) position at DPD UK. On their website, DPD UK describes this position as follows:

If you’re looking for a full time owner operator business opportunity with low startup costs, then the Owner Driver Franchise (ODF) is for you. As an ODF, you’ll provide collection and delivery services to DPD typically over five days per week, with flexibility for you to choose the days that fit your lifestyle. We operate seven days a week, which means there’s opportunities for you to increase your earnings further (DPD 2021).

The parcel deliverers are not only promised flexibility, but also high earnings. Within 3 years, they can earn a turnover of as much as £170,000, which is, according to DPD UK, “dependent on you delivering fantastic collection and delivery service but totally achievable”. Drivers have the freedom to take on additional routes, which increases their earnings. Furthermore, on the website of DPD UK, drivers report positively about their increased work autonomy and flexibility, as shows from the quotes: “I’m my own boss, I love the freedom of being on the road” and “I now have a much better work/home life balance.”

A similar message can be found on the website of Amazon Flex, Amazon’s version of the self-employed courier platform service (Amazon 2021). The recruitment slogan for Amazon Flex contains a clear promise of self-realization: “No matter what your goal is, Amazon Flex helps you get there.” Also, the ideal of flexibility clearly appears as one of the promises made to future Amazon Flex couriers:

We know how valuable your time is. With Amazon Flex, you work only when you want to. You can plan your week by reserving blocks in advance or picking them each day based on your availability. Choose the blocks that fit your schedule, then get back to living your life.

As a *second step*, a normative reconstruction of platform work requires the identification of institutional practices that differentially affect the realization of these norms, values and promises surrounding work in the platform economy. In this respect, the available empirical evidence on the material conditions of platform work provides meaningful insights. An increasing body of reports and academic studies lays bare the precarious working conditions of platform work, that in practice deny many platform workers the realization of the promises of flexibility and autonomy that the platform model contains (Berg 2016; Berg et al. 2018; Brancati et al. 2020; Huws et al. 2017; Joyce et al. 2020). Platform workers, for instance, appear to earn a lower wage on average than workers in non-platform working situations, especially when including non-paid working time (e.g. time in between platform tasks or searching for new tasks on the platform). Moreover, they tend to work longer (unsocial) hours, resulting in less time to spend with family or friends. Also, platform workers seem to face highly unstable demands for work and suffer more health issues (Huws et al. 2017; Joyce et al. 2020). Although the findings vary for different platform sectors, the common denominator of most empirical studies on platform work is the conclusion that platform workers often find themselves in a more precarious working position, compared to non-platform workers.

The platform business model, in addition, contains several characteristics that constrain the independence of platform workers. First, platform workers tend to occupy a position of contractual weakness vis-à-vis large digital platforms, which often results in abusive contractual conditions (Dononi et al. 2017). Since platform workers are self-employed and thus qualify, legally and economically, as enterprises, they enter a contractual relationship with the platform based on presumed equality. Such equality, however, hardly exists in reality, where the economic scale of large digital platforms enables the latter to unilaterally control and modify the terms and conditions under which platform work is performed. The example of Uber, which centrally determines the prices for rides through its surge pricing algorithm, is illustrative in this regard (Chen and Sheldon 2015). Other examples of abusive practices are the refusal to provide transparency with regard to the fundamental conditions of the work, such as the determination of payments or working schedules. Some platforms have strict rules for missing or not performing assigned work, which can result in high penalty payments or exclusion from the platform.

Second, the platform business model has been criticized as a real-world testing ground for so-called “algorithmic management” of workers (Duggan et al. 2019; Möhlmann et al. 2020). This algorithmic management has been described as “a diverse set of technological tools and techniques to remotely manage workforces, relying on data collection and surveillance of workers to enable automated or semi-automated decision-making” (Mateescu and Nguyen 2019: 3)*.* The systems are designed to collect data from workers and monitor their performance (either by the system itself or via user rating systems) and either nudge workers to increase efficiency or punish their failure to meet targets. Such systems have been directly criticized for their reduction of the voluntary and flexible nature of platform work: “algorithmic systems can use a variety of methods to structure and control worker behavior, even when the platforms hosting those systems are billed as flexible or voluntary” (Mateescu and Nguyen 2019: 13)*.* As a result of these systems, workers experience speed and efficiency pressures, since these systems are not programmed to take unforeseen circumstances into account.

The discrepancy between espoused values and material working conditions equally appears in the practices of platforms like DPD and Amazon Flex. In recent years, the Owner Driver Franchise working position at DPD UK has not been spared of controversy. In 2017, UK MP Frank Field published a report revealing the dreadful working conditions of DPD UK parcel couriers (Field 2017). Drivers allegedly earned hourly wages as low as £2.50, faced threatening and hostile working environments and faced charges of £150 when they missed a shift and were unable to find a replacement driver. Following the report, the Guardian interviewed parcel drivers, who felt exploited and undervalued:

While profits soar, we as drivers get exploited, underpaid and taken for granted… Conditions of employment are precarious to say the least. The communication received from DPD always has a threatening edge to it as they know people worry about their jobs… If you work at the same depot every day, wear a DPD uniform, drive a DPD van, get given a DPD scanner, are told by DPD management what parcels to deliver at what rate, don’t work for anyone else, you legally should be protected by the government as being employed… This isn’t self-employment, or employment either. It’s a living hell, a nightmare scenario and the government needs to bring in legislation to stop these crooks from ripping off vulnerable people (Davies and Butler 2017).

When in January 2018, a DPD courier died after missing hospital appointments for diabetes – reportedly because he was terrified to take time off – public outrage led DPD to remove the £150 fine clause from the contracts with couriers and to offer all couriers the option to enter a position as employee at DPD (Booth 2018).

Amazon Flex has likewise been the subject of sharp criticism. Reportedly, it is not uncommon for Flex couriers to drive more than 11 h consecutively (which is against UK laws), to receive net earnings that can fall below minimum wage levels and to be fined for missing targets (Del Valle 2018). Furthermore, the previously discussed algorithmic management devices are, according to a former driver, designed to force couriers to drive quickly, which inevitable results in hazardous situations and potential damages – the repairs of which the self-employed couriers have to pay themselves. In addition, Amazon has admitted to covert surveillance of its Flex couriers’ online activities, by monitoring private Facebook groups and public forums, which have to report on discussions regarding the working conditions of Flex couriers. News website The Verge reports as follows:

Amazon also appears to be keeping tabs on more sensitive discussions. Those compiling the reports are instructed to note the apparent sentiment of posts and to look for Flex workers sharing news stories where “warehouse employees [are] complaining about the poor working condition” or that discuss “planning for any strike or protest against Amazon” (Vincent 2020).

This section illustrates how the powerful bargaining position of digital platforms vis-à-vis individual workers enables the reduction of the flexibility and autonomy that platform workers experience and force platform workers into a straightjacket of hyper productivity. A normative paradox thus emerges: while the digital platform model promises an environment where flexibility, self-management and autonomy is the norm, it allows platforms to influence material working conditions in such a way as to prevent the realization of these ideals. The model thus misrecognizes the skewed distribution of economic power between the worker and the platform, providing platforms a *carte blanche* to exploit this position of relative weakness of the worker. With Honneth, one could classify these promises as merely ideological, in the sense that their function is to foster productivity growth and uphold existing power structures. Honneth points to the inherent inability of workers to realize the ideals of flexibility and autonomy in their work under such conditions: “the new manner of addressing employees and qualified workers as entrepreneurs of their own labor power might contain an evaluative promise of recognizing a higher degree of individuality and initiative, but it in no way ensures the institutional measures that would allow a consistent realization of these new values” (Honneth 2007a: 346).

## Discussion and Conclusions

In this paper, we have extended Honneth’s theory to an analysis and critique of working conditions in the platform economy. By employing his method of normative reconstruction, we were able to discern a normative paradox in the ways platform organizations function, promising flexibility and autonomy while at the same time creating working conditions that undercut these promises. While thus seemingly applicable to the neoliberal workplace as exemplified in the platform economy and while popular in MOS, Honneth’s theory has not been precluded from critique.

It has been characterized as a “perfectionist” or “positive” model of recognition, in which a lack of recognition or negative forms of recognition, as experienced or perceived by certain societal groups, are remedied by either extending or reorienting existing patterns of recognition so as to fill the recognitive void (Klikauer 2016; Lepold 2019; McQueen 2015). As such, it has been critiqued, in the first place, for overlooking “the ways in which recognition functions to produce viable and unviable identities; and is intertwined with processes of exclusion and normalization” (McQueen 2015: 45–46; Sebrechts et al. 2019). From this perspective, which draws on French philosophers like Althusser and Foucault and is currently most clearly visible in the work of Judith Butler, recognition is regarded as a condition that enables *and* constrains freedom at the same time (Bertram and Celikates 2015; Ikäheimo 2017). By recognizing B as a subject, A confers freedom on B, but at the same time subjectivizes B, i.e. makes B dependent on A (and vice versa).

Honneth has responded to this critique in a discussion of Althusser, for whom “individuals can become socially identifiable subjects only by being subjected through public recognition to a web of social rules that does not possess any room for variation with respect to individual autonomy” (Honneth 2007a: 330–331). While acknowledging that human subjectivity can be influenced by pre-existing systems of discourse and social norms, Honneth does not adhere to the view that these norms fully “invalidat[e] the idea of autonomy in the sense of the authorship of the subject,” as Althusser seems to imply; instead, Honneth seeks to develop “a theory of intersubjectivity” that is able to account for “subject-transcending powers as constitutive conditions for individualization of subjects” (Honneth 2007c: 181, 183).

At the same time, Honneth seems mindful of the double-sided nature of recognition. His ideas on “organized self-realization” and “recognition as ideology” (Honneth 2004, 2007a) point at discourses that seem to fulfill normative expectations and thus to fill recognitive voids, but at the same time are realized in such a way as to thwart these discursive promises. At least in a sociological and psychological sense, this comes close to recognition as enabling and constraining at the same time, i.e. as some form of double bind: in *Sorry We Missed You* Ricky is “the master of his own destiny,” although that destiny is fully determined by others.

In the second place, Honneth’s method of normative reconstruction has been subject of critique. This method presupposes that norms and promises of self-realization and positive identity formation are immanently present in different spheres of society (including the market sphere), taking these “immanently justified values as a criterion for processing and sorting out the empirical material” (Honneth 2014: 6). This presupposition has been critiqued as presenting a too optimistic view of the possibility of such norms and promises, or even “normative surpluses,” under market conditions, in particular under the current neoliberal capitalism that is intent on subverting these surpluses (Buchwalter 2017; Carleheden 2020; Fazio 2019; Jütten 2015). The “drying up of normative surpluses” would deprive Honneth’s approach from both its critical edge, “destroy[ing] the radical needs whose demands for satisfaction had pointed beyond the established social order,” and its sociological dimension, threatening a return to previous Frankfurt grand metanarratives (Johnson 2014: 527). A similar pessimism has even led to a small-scale “post-recognition” turn in MOS, according to which it is no longer rational or feasible to struggle for recognition within a neoliberal capitalist system that is quite capable of absorbing these struggles and other forms of resistance. Instead, the best option is to “exit” that system and to resort to autonomy, invisibility and anonymity inside organizations (Fleming 2016; Mumby et al. 2017) and in the (digital) world at large (Odell 2019; Zuboff 2015).

However, an answer to the “post-recognition” critique can be found in Honneth’s own philosophical sources. Much of his thinking about the new “entreployees” is based on the work of Boltanski and Chiapello (2018), to whom he regularly refers in his “paradox papers” (Hartmann and Honneth 2006: 45, 50, 56; Honneth 2004: 473–474; Honneth and Sutterlüty 2011: 75–76), and whose central proposition is that capitalism, defined as “the imperative to unlimited accumulation of capital by formally peaceful means,” is by itself an unattractive and absurd system: not only does it deprive workers of the fruits of their labor in a relationship of subservience to their employers, but it also chains the capitalists themselves to an interminable and insatiable process of accumulation (Boltanski and Chiapello 2018: 4, 7). Such a system is continuously in need of a “spirit of capitalism,” i.e. an ideology that serves to justify people’s enthusiasm for and trust in the capitalist system, even though not many will be able to fully share in its profits.

From this proposition it follows that there always will be normative promises and expectations through which the capitalist system attempts to showcase its attractiveness, and which then can be put to the test of normative reconstruction, both at state and firm levels. At the firm level, for example, this enables an analysis of how organizations portray internal employment and working conditions in their public communications (websites, reports, advertisements, etc.), followed by an empirical investigation of the actual employment and working conditions in these organizations and the degree to which they support the espoused conditions (Visser 2019; Willmott 2020); such an analysis would in fact follow the story line of *Sorry We Missed You*, as the examples of DPD and Amazon Flex show.

Further, an answer to the “post-recognition” critique can be found in already existing institutional and legal developments stimulating the creation of a platform working environment that respects the normative promises of flexibility and autonomy. One development is the strengthening of platform worker representation and organization. Unions increasingly appear to be open to including platform workers in their collective bargaining efforts, while new grassroots and digital counter movements such as Turkopticon contribute to the formation of a counterbalance against the market power of large digital platforms (Benassi and Vlandas 2016; Lenaerts et al. 2018; Vandaele 2020). Another development can be found in the field of labor law, where EU member states are experimenting with the creation of intermediate legal categories for platform workers, which recognize their position as somewhere in between a full-fledged worker and a solo self-employed (Laagland and Kloostra 2019). In the field of competition law, increased attention is devoted to the protection of small businesses and platform suppliers against unfair contractual terms imposed by digital platforms. In 2019, the European Commission adopted the Platform-to-Business (P2B) regulation, which attempts to prevent unfair contractual obligations regarding, among others, suspension of platform workers, discriminatory treatment and harmful clauses concerning ranking and review mechanisms.

All in all, while not without its critics and supplemented by the work of Boltanski and Chiapello, it appears that Honneth’s recognition theory and method of normative reconstruction go a long way in analyzing and critiquing current neoliberal work practices, as exemplified here in the platform economy. In principle, his theory and approach could be applied to various other work and organizational phenomena, where reasonable suspicions may be held that normative expectations and promises are not supported or even eclipsed by organizational or institutional practices. One example could be corporate social responsibility, with which in particular private sector firms showcase their social and ecological responsibilities beyond the “bottom line,” but which they often contradict with less “responsible” practices like tax evasion and pollution (e.g., Schneider 2020). Another example could be gender diversity, with which firms showcase their awareness of diversity and inclusion, but which they often contradict with masculine work and promotion practices (e.g., Edgley et al. 2016). Current neoliberal organizations seem full of normative promises and contradictions, and are likely to remain so as the world braces for post-Covid-19 economic impact.

## References

[CR1] Aktouf O (1992). Management and theories of organizations in the 1990s: Toward a critical radical humanism?. Academy of Management Review.

[CR2] Alvesson M, Willmott H (1992). On the idea of emancipation in management and organization studies. Academy of Management Review.

[CR3] Alvesson, M., and H. Willmott. 2012. *Making sense of management: A critical introduction*. 2nd ed. London: Sage.

[CR4] Amazon. 2021. https://flex.amazon.com/. Accessed 9 Jan 2021.

[CR5] Anderson, J. 2011. Situating Axel Honneth in the Frankfurt school tradition. In *Axel Honneth: Critical essays*, ed. D. Petherbridge, 31–57. Leiden/Boston: Brill.

[CR6] Bailey C, Lips-Wiersma M, Madden A, Yeoman R, Thompson M, Chalofsky N (2019). The five paradoxes of meaningful work: Introduction to the special issue “meaningful work: Prospects for the 21st century”. Journal of Management Studies.

[CR7] Benassi C, Vlandas T (2016). Union inclusiveness and temporary agency workers: The role of power resources and union ideology. European Journal of Industrial Relations.

[CR8] Berg J (2016). Income security in the on-demand economy: Findings and policy lessons from a survey of crowd workers. Comparative Labor Law and Policy Journal.

[CR9] Berg, J., M. Furrer, E. Harmon, U. Rani, and M.S. Silberman. 2018. *Digital labor platforms and the future of work: Towards decent work in the online world*. Geneva: International Labor Office.

[CR10] Bertram GW, Celikates R (2015). Towards a conflict theory of recognition: On the constitution of relations of recognition in conflict. European Journal of Philosophy.

[CR11] Boltanski, L., and E. Chiapello. 2018. [1999]*. The new spirit of capitalism (new updated ed.) (transl. G. Elliott)*. London: Verso.

[CR12] Booth, R. 2018. DPD courier who was fined for day off to see doctor dies from diabetes. The Guardian*.* https://www.theguardian.com/business/2018/feb/05/courier-who-was-fined-for-day-off-to-see-doctor-dies-from-diabetes. Accessed 12 Jan 2021.

[CR13] Brancati, U., A. Pesole, and E. Férnandéz-Macías. 2020. New evidence on platform workers in Europe. In *Results from the second COLLEEM survey*. Luxembourg: Publications Office of the European Union.

[CR14] Buchwalter, A. 2017. The concept of normative reconstruction: Honneth, Hegel, and the aims of critical social theory. In *Reconstructing social theory, history and practice (Current Perspectives in Social Theory, Vol. 35)*, ed. H.F. Dahms and E.R. Lybeck, 57–88. Bingley: Emerald.

[CR15] Carleheden, M. 2020. How to criticize? On Honneth’s method. *Distinktion: Journal of Social Theory*. 10.1080/1600910X.2020.1779100.

[CR16] Carson CM (2005). A historical view of Douglas McGregor’s theory Y. Management Decision.

[CR17] Chen, M., and M. Sheldon. 2015. *Dynamic pricing in a labor market: Surge pricing and the supply of Uber driver-partners*. UCLA Anderson https://citeseerx.ist.psu.edu/viewdoc/download?doi=10.1.1.704.3600&rep=rep1&type=pdf. Accessed 12 Jan 2021.

[CR18] Connolly J (2016). Honneth on work and recognition: A rejoinder from feminist political economy. Thesis Eleven.

[CR19] Curty G (2020). Capitalism, critique and social freedom: An interview with Axel Honneth on Freedom’s right. Critical Sociology.

[CR20] Davies W (2016). The new neoliberalism. New Left Review.

[CR21] Davies, R. and S. Butler. 2017. UK workers earning £2.50 an hour prompts call for government action. The Guardian. https://www.theguardian.com/business/2017/jul/06/uk-workers-poverty-pay-gig-economy-frank-field-report. Accessed 12 Jan 2021.

[CR22] Del Valle, G. 2018. *Amazon is cutting costs with its own delivery service — But its drivers don’t receive benefits*. Vox https://www.vox.com/the-goods/2018/12/26/18156857/amazon-flex-workers-prime-delivery-christmas-shopping. Accessed 12 Jan 2021.

[CR23] Dononi A, Forlivesi M, Rota A, Tullini P (2017). Towards collective protections for crowd workers: Italy, Spain and France in the EU context. Transfer.

[CR24] DPD. 2021. Owner driver franchise opportunities. https://drivers.dpd.co.uk/being-an-odf. Accessed 9 Jan 2021.

[CR25] Drahokoupil J, Piasna A (2017). Work in the platform economy: Beyond lower transaction costs. Intereconomics.

[CR26] Duggan J, Sherman U, Carbery R, McDonnell A (2019). Algorithmic management and app-work in the gig economy: A research agenda for employment relations and HRM. Human Resource Management Journal.

[CR27] Edgley C, Sharma N, Anderson-Gough E (2016). Diversity and professionalism in the big four firms: Expectation, celebration and weapon in the battle for talent. Critical Perspectives on Accounting.

[CR28] Evans DS (2008). Competition and regulatory policy for multi-sided platforms with applications to the web economy. Concurrences.

[CR29] Fazio G (2019). From Hegel to Foucault and back? On Axel Honneth’s interpretation of neoliberalism. Philosophy and Social Criticism.

[CR30] Field, F. 2017. A new contract for the ‘gig economy’ Britain’s new self-employed at BCA, DPD and Parcelforce. http://www.frankfield.co.uk/upload/docs/A%20new%20contract%20for%20the%20%27gig%20economy%27.pdf. Accessed 12 Jan 2021.

[CR31] Fleming P (2016). Resistance and the “post-recognition” turn in organizations. Journal of Management Inquiry.

[CR32] Hartmann M, Honneth A (2006). Paradoxes of capitalism. Constellations.

[CR33] Helmond A (2015). The platformization of the web: Making web data platform ready. Social Media + Society.

[CR34] Holtgrewe U (2001). Recognition, intersubjectivity and service work: Labor conflicts in call centers. Industrielle Beziehungen.

[CR35] Honneth, A. 1995. *The struggle for recognition: The moral grammar of social conflicts (transl. J. Anderson)*. Cambridge: Polity.

[CR36] Honneth A (2004). Organized self-realization: Some paradoxes of individualization. European Journal of Social Theory.

[CR37] Honneth, A. 2007a. Recognition as ideology. In *Recognition and power: Axel Honneth and the tradition of critical social theory*, ed. B. van den Brink and D. Owen, 323–347. Cambridge: Cambridge University Press.

[CR38] Honneth, A. 2007b. Between Aristotle and Kant: Recognition and moral obligation. In *Disrespect: The normative foundations of critical theory*, 129–143. Cambridge: Polity.

[CR39] Honneth, A. 2007c. Decentered autonomy: The subject after the fall. In *Disrespect: The normative foundations of critical theory*, 181–193. Cambridge: Polity.

[CR40] Honneth, A. 2008. Reification and recognition: A new look at an old idea. In *Reification: A new look at an old idea*, ed. M. Jay, 17–94. Oxford: Oxford University Press.

[CR41] Honneth, A. 2014. *Freedom’s right: The social foundations of democratic life (transl. J. Ganahl)*. New York: Columbia University Press.

[CR42] Honneth A, Sutterlüty F (2011). Normative Paradoxien der Gegenwart: Eine Forschungsperspektive. WestEnd: Neue Zeitschrift für Sozialforschung.

[CR43] Huws, U., N.H. Spencer, D.S. Syrdal, and K. Holts. 2017. *Work in the European gig economy*. Brussels: Foundation For European Progressive Studies.

[CR44] Ikäheimo, H. 2017. Recognition, identity and subjectivity. In *Palgrave handbook of critical theory*, ed. M.J. Thompson, 567–585. New York: Palgrave Macmillan.

[CR45] Islam G (2012). Recognition, reification, and practices of forgetting: Ethical implications of human resource management. Journal of Business Ethics.

[CR46] Islam G (2013). Recognizing employees: Reification, dignity and promoting care in management. Cross Cultural Management.

[CR47] Johnson P (2014). Sociology and the critique of neoliberalism: Reflections on Peter Wagner and Axel Honneth. European Journal of Social Theory.

[CR48] Joyce S, Stuart M, Forde C, Valizade D (2020). Work and social protection in the platform economy in Europe. Advances in Industrial and Labor Relations.

[CR49] Jullien B (2005). Two-sided markets and electronic intermediaries. CESifo Economic Studies.

[CR50] Jütten T (2015). Is the market a sphere of social freedom?. Critical Horizons.

[CR51] Keucheyan, R. 2014. *The left hemisphere: Mapping critical theory today (transl. G. Elliott)*. London: Verso.

[CR52] Klikauer T (2016). Negative recognition: Master and slave in the workplace. Thesis Eleven.

[CR53] Laagland F, Kloostra J (2019). De Engelse tussencategorie als oplossing voor platformwerk: Mythe of werkelijkheid?. Ondernemingsrecht.

[CR54] Lenaerts K, Kilhoffer Z, Akgüc M (2018). Traditional and new forms of organization and representation in the platform economy. Digital Economy and the Law.

[CR55] Lepold K (2019). Examining Honneth’s positive theory of recognition. Critical Horizons.

[CR56] Lussier K (2019). Of Maslow, motives, and managers: The hierarchy of needs in American business, 1960-1985. Journal of the History of the Behavioral Sciences.

[CR57] Maslow, A.H. 1965. *Eupsychian management: A journal*. Homewood: Irwin & Dorsey Press.

[CR58] Mateescu, A., and A. Nguyen. 2019. Explainer: Algorithmic management in the workplace. Data and Society*.* https://datasociety.net/wp-content/uploads/2019/02/DS_Algorithmic_Management_Explainer.pdf. Accessed 12 Jan 2021.

[CR59] McGregor, D.M. 1960. *The human side of enterprise*. New York: McGraw-Hill.

[CR60] McQueen P (2015). Honneth, Butler and the ambivalent effects of recognition. Res Publica.

[CR61] Möhlmann, M., L. Zalmanson, O. Henfridsson, and R.W. Gregory. 2020. Algorithmic management of work on online labor platforms: When matching meets control. *MIS Quarterly* https://misq.org/forthcoming/.

[CR62] Mokatef M (2019). Recognition and precarity of life arrangement: Towards an enlarged understanding of precarious working and living conditions. Distinktion: Journal of Social Theory.

[CR63] Mumby DK, Thomas R, Martí I, Seidl D (2017). Resistance redux: Introduction to the special issue. Organization Studies.

[CR64] Odell, J. 2019. *How to do nothing: Resisting the attention economy*. New York: Melville House.

[CR65] Outhwaite W (2009). Recognition, reification and (dis)respect. Economy and Society.

[CR66] Parker GG, Van Alstyne MW (2005). Two-sided network effects: A theory of information product design. Management Science.

[CR67] Peck J (2013). Explaining (with) neoliberalism. Territory, Politics, Governance.

[CR68] Petersen A, Willig R (2004). Work and recognition: Reviewing new forms of pathological developments. Acta Sociologica.

[CR69] Prud’homme, D. 2019. How digital businesses can leverage the high cost for consumers to switch platforms. *London School of Economics Business Review* https://blogs.lse.ac.uk/businessreview/2019/09/24/how-digital-businesses-can-leverage-the-high-cost-for-consumers-to-switch-platforms/. Accessed 12 Jan 2021.

[CR70] Rochet J, Tirole J (2006). Two-sided markets: A progress report. The Rand Journal of Economics.

[CR71] Rysman M (2009). The economics of two-sided markets. Journal of Economic Perspectives.

[CR72] Scherer, A.S. 2009. Critical theory and its contribution to critical management studies. In *Oxford handbook of critical management studies*, ed. M. Alvesson, T. Bridgman, and H. Willmott, 29–51. Oxford: Oxford University Press.

[CR73] Schneider A (2020). Bound to fail? Exploring the systemic pathologies of CSR and their implications for CSR research. Business & Society.

[CR74] Sebrechts ME, Tonkens E, Da Roit B (2019). Unfolding recognition: An empirical-theoretical contribution to the concept. Distinktion: Journal of Social Theory.

[CR75] Tweedie D (2017). The normativity of work: Retrieving a critical craft norm. Critical Horizons.

[CR76] Tweedie D (2018). After Habermas: Applying Axel Honneth’s critical theory in accounting research. Critical Perspectives on Accounting.

[CR77] Tweedie D, Holley S (2016). The subversive craft worker: Challenging “disutility” theories of management control. Human Relations.

[CR78] Tweedie D, Wild D, Rhodes C, Martinov-Bennie N (2019). How does performance management affect workers? Beyond human resource management and its critique. International Journal of Management Reviews.

[CR79] Vandaele, K. 2020. Collective resistance and organizational creativity amongst Europe’s platform workers: A new power in the labor movement? *Work and Labor Relations in Global Platform Capitalism*. 10.2139/ssrn.3672260.

[CR80] Varul MZ (2005). Marx, morality and management: The normative implications of the labor value theory and the contradictions of HRM. Philosophy of Management.

[CR81] Vincent, J. 2020. *Amazon is reportedly surveilling its flex delivery drivers in private Facebook groups*. The Verge https://www.theverge.com/2020/9/2/21418057/amazon-surveilling-flex-delivery-drivers-facebook-groups-subreddits-strikes-protests. Accessed 12 Jan 2021.

[CR82] Visser M (2019). Pragmatism, critical theory and business ethics: Converging lines. Journal of Business Ethics.

[CR83] Voswinkel S (2011). Paradoxien entgrenzter Arbeit. WestEnd: Neue Zeitschrift für Sozialforschung.

[CR84] Willmott H (2020). On research methodology. Journal of Organization and Discourse.

[CR85] Wismer S, Bongard C, Rasek A (2017). Multi-sided market economics in competition law enforcement. Journal of European Competition Law & Practice.

[CR86] Zhu F, Iansiti M (2019). Why some platforms thrive and others don’t. Harvard Business Review.

[CR87] Zuboff S (2015). Big other: Surveillance capitalism and the prospects of an information civilization. Journal of Information Technology.

[CR88] Zurn, C.F. 2015. *Axel Honneth: A critical theory of the social*. Cambridge: Polity.

